# Comparative Evaluation of Salivary Inflammatory Cytokines and microRNA Biomarkers Across the Spectrum of Oral Carcinogenesis

**DOI:** 10.7759/cureus.112931

**Published:** 2026-07-18

**Authors:** Beena George, Shital Kharat, Roopan Prakash, Omkar Eswara B Danda, Rachna Raj, Chintan M Upadhyay, Sajidali S Saiyad

**Affiliations:** 1 Oral Pathology and Microbiology, Sree Mookambika Institute of Dental Sciences, Kulasekharam, IND; 2 Oral Pathology and Microbiology, Government Dental College, Jalgaon, IND; 3 Oral Pathology and Microbiology, Eklavya Dental College, Kotputli, IND; 4 Conservative Dentistry and Endodontics, Dr NTR University of Health Sciences, Vijayawada, IND; 5 Public Health Dentistry, Patna Dental College and Hospital, Patna, IND; 6 Obstetrics and Gynecology, Dr. ND Desai Faculty of Medical Science and Research, Dharmsinh Desai University, Nadiad, IND; 7 Physiology, Pacific Medical College and Hospital, Pacific Medical University, Udaipur, IND

**Keywords:** liquid biopsy, microrna, mir-138, mir-184, mir-21, oral potentially malignant disorders, oral squamous cell carcinoma, precision oncology, salivary biomarkers

## Abstract

Background

Oral potentially malignant disorders (OPMDs) represent precursor conditions that carry an elevated probability of progressing to oral squamous cell carcinoma (OSCC). Despite ongoing research efforts, identifying dependable, non-invasive markers that can detect early molecular changes linked to malignant conversion continues to present a significant clinical obstacle.

Objective

To evaluate the diagnostic utility of salivary inflammatory cytokines (IL-10 and IL-17) and microRNAs (miR-21, miR-138, and miR-184) in healthy individuals, patients with OPMDs, and patients with OSCC, and to assess the performance of a combined biomarker panel for oral cancer clinical stratification.

Methods

A cross-sectional analytical study was conducted involving 150 participants categorized into healthy controls (n=50), OPMD patients (n=50), and histopathologically confirmed OSCC patients (n=50). Salivary IL-10 and IL-17 concentrations were quantified using enzyme-linked immunosorbent assay (ELISA), whereas miR-21, miR-138, and miR-184 expression levels were measured using quantitative reverse-transcription polymerase chain reaction (qRT-PCR). Relative miRNA expression was calculated using the 2^−ΔΔCt method. Diagnostic performance was evaluated using receiver operating characteristic (ROC) curve analysis.

Results

Significant alterations in salivary cytokine and microRNA expression were observed across the disease spectrum. miR-21 and miR-184 demonstrated progressive upregulation, whereas miR-138 showed progressive downregulation from healthy controls to OPMD and OSCC patients (all p<0.001). Both IL-10 and IL-17 were significantly elevated in OPMD and OSCC patients compared with healthy controls (both p<0.001). Biomarker expression profiles were significantly associated with dysplasia severity and tumor stage. Among individual biomarkers, miR-138 demonstrated the highest diagnostic accuracy (AUC=0.84), followed by IL-17 (AUC=0.82), miR-21 (AUC=0.80), miR-184 (AUC=0.76), and IL-10 (AUC=0.74). The combined cytokine-microRNA panel achieved superior performance, with an AUC of 0.93, sensitivity of 90%, and specificity of 88%.

Conclusions

The findings support the potential utility of integrated salivary biomarker profiling as a non-invasive approach for early detection, clinical stratification, and disease monitoring in oral carcinogenesis. The combined biomarker panel highlights the potential of integrated salivary diagnostics for improving the non-invasive detection and clinical assessment of OPMDs and OSCC.

## Introduction

Oral potentially malignant disorders (OPMDs) encompass a diverse collection of oral mucosal conditions that carry an elevated likelihood of transforming into oral squamous cell carcinoma (OSCC), which represents the predominant malignant neoplasm affecting the oral cavity [1]. Frequently encountered OPMDs include leukoplakia, lichen planus, erythroplakia, and submucous fibrosis. While many of these lesions remain clinically stable over time, a subset undergoes progressive molecular and cellular changes involving persistent inflammatory responses, oxidative damage, genetic instability, epigenetic modifications, and tumor microenvironment alterations, ultimately culminating in malignant conversion [1,2].

Despite therapeutic and diagnostic progress, OSCC continues to impose substantial morbidity and mortality globally. Patient outcomes correlate closely with diagnostic stage, yet a considerable number of cases are still detected at advanced stages. Although histopathology remains the established diagnostic benchmark, tissue biopsy is invasive, prone to sampling errors, and impractical for serial monitoring. Furthermore, histopathology alone cannot reliably predict which OPMDs will undergo malignant transformation, highlighting the need for non-invasive biomarkers capable of identifying early molecular alterations associated with oral carcinogenesis [1-3].

Saliva has gained recognition as a valuable diagnostic fluid due to its non-invasive collection, repeatability, and cost-effectiveness. Owing to its direct contact with oral lesions, saliva contains disease-associated biomolecules that reflect local pathological changes. Recent systematic reviews have demonstrated the potential utility of salivary proteins, metabolites, cytokines, nucleic acids, and extracellular vesicle-derived molecules for the detection and monitoring of OPMDs and OSCC [4,5].

Among these biomarkers, inflammatory cytokines have attracted considerable attention because chronic inflammation is a hallmark of oral carcinogenesis. Persistent inflammatory signaling promotes epithelial proliferation, angiogenesis, immune dysregulation, and genomic instability [2,6]. Several cytokines, including interleukin (IL)-10 and IL-17, exhibit altered salivary concentrations in OPMD and OSCC patients. IL-17 contributes to a pro-tumorigenic inflammatory microenvironment, whereas IL-10 facilitates immune suppression and tumor immune escape. Previous studies have reported significant alterations in salivary IL-10 and IL-17 levels, supporting their potential value as diagnostic and prognostic biomarkers [6,7].

MicroRNAs (miRNAs) represent another promising biomarker class. These small non-coding RNAs regulate gene expression post-transcriptionally and play critical roles in cellular proliferation, differentiation, apoptosis, invasion, and metastasis. Dysregulated miRNA expression has been consistently reported during the progression from OPMDs to OSCC. Recent evidence highlights miR-21, miR-138, and miR-184 as clinically relevant salivary biomarkers. miR-21 functions predominantly as an oncogenic miRNA, whereas miR-138 and miR-184 are involved in pathways regulating epithelial differentiation and malignant progression [3,8,9].

Emerging evidence suggests that inflammatory cytokines and miRNAs are biologically interconnected during oral carcinogenesis. Inflammatory signaling can modulate miRNA expression, while dysregulated miRNAs influence cytokine production and immune responses. Consequently, simultaneous evaluation of inflammatory and epigenetic biomarkers may provide greater diagnostic accuracy than either biomarker class alone.

Although substantial evidence supports the diagnostic utility of salivary cytokines and miRNAs individually, studies integrating both biomarker classes within a single diagnostic framework remain limited. Therefore, the present study compared salivary IL-10, IL-17, miR-21, miR-138, and miR-184 levels among healthy individuals, patients with OPMDs, and patients with OSCC. Additionally, the diagnostic performance of individual biomarkers and integrated cytokine-miRNA panels was evaluated to explore their potential utility in the early detection and risk stratification of oral malignant transformation.

## Materials and methods

Study design and ethical approval

This hospital-based cross-sectional analytical study examined the expression profiles and diagnostic value of selected salivary inflammatory cytokines and microRNAs (miRNAs) in healthy individuals, patients with oral potentially malignant disorders (OPMDs), and patients with oral squamous cell carcinoma (OSCC). The study protocol received approval from the Institutional Ethics Committee of Pacific Medical College and Hospital, Udaipur (Approval No. PMU/PMCH/IEC/GEN/2023/96, dated 10 June 2023). Written informed consent was secured from all participants prior to their enrollment.

Study population

A total of 150 participants were recruited and divided into three equal groups: healthy controls (n = 50), patients with OPMDs (n = 50), and patients with histopathologically confirmed OSCC (n = 50). OPMD diagnoses included oral leukoplakia, oral submucous fibrosis, oral lichen planus, and erythroplakia, established through clinical examination and histopathological confirmation when indicated. OSCC patients were treatment-naïve with biopsy-verified disease.

Participants aged 18 years and above were eligible. Healthy controls had no history of oral mucosal lesions, malignancy, or chronic inflammatory conditions. Exclusion criteria included acute oral infections, severe periodontal disease, autoimmune disorders, diabetes mellitus, other malignancies, prior chemotherapy or radiotherapy, immunosuppressive therapy, pregnancy, lactation, or unwillingness to provide saliva samples.

Clinical and histopathological assessment

Demographic and clinical data, including age, sex, tobacco use, smoking status, areca nut consumption, alcohol intake, lesion duration, and lesion site, were collected using a structured proforma. For OPMD patients, lesion type and histopathological grade of epithelial dysplasia were recorded. For OSCC cases, tumor site, histological differentiation, and clinical stage were documented according to standard criteria.

Saliva collection and processing

To reduce potential circadian influences, unstimulated saliva specimens were obtained during the morning period (08:00-10:00). [4,6]. Participants abstained from eating, drinking, smoking, chewing tobacco, and oral hygiene procedures for at least one hour prior to collection. After rinsing the mouth with distilled water and resting for five minutes, approximately 5 mL of saliva was obtained by passive drooling into sterile polypropylene tubes.

Following collection, specimens were promptly transferred to the laboratory under refrigerated conditions. To eliminate cellular components, samples were subjected to centrifugation (2600×g, 15 min, 4°C). The resulting supernatant was distributed into RNase-free tubes and preserved at -80°C pending analysis.

Quantification of salivary cytokines

Salivary IL-10 and IL-17 concentrations were measured using commercially available human enzyme-linked immunosorbent assay (ELISA) kits (Elabscience Biotechnology Co., Ltd., Wuhan, China) according to the manufacturer's instructions. Samples and standards were run in duplicate. Absorbance was recorded at 450 nm using a Bio-Rad iMark™ Microplate Reader (Bio-Rad Laboratories, Hercules, CA, USA). Concentrations were calculated from standard calibration curves and expressed in picograms per milliliter (pg/mL).

microRNA extraction and reverse transcription

RNA extraction, encompassing small RNA species, was performed on saliva supernatant using the miRNeasy Mini Kit (QIAGEN, Hilden, Germany) according to the manufacturer's prescribed protocol. RNA concentration and purity were evaluated with a NanoDrop™ 2000 spectrophotometer (Thermo Fisher Scientific, Wilmington, DE, USA). Only samples with an A260/A280 ratio of 1.8-2.1 were used for downstream analysis. Complementary DNA (cDNA) was synthesized using the miScript II RT Kit (QIAGEN, Hilden, Germany) as per manufacturer guidelines.

Quantitative real-time polymerase chain reaction

Expression of miR-21, miR-138, and miR-184 was quantified by quantitative real-time polymerase chain reaction (qRT-PCR) on a QuantStudio™ 5 Real-Time PCR System (Applied Biosystems, Thermo Fisher Scientific, USA). miR-16 served as the endogenous reference control. All reactions were performed in duplicate, and mean cycle threshold (Ct) values were used for analysis. The thermal cycling conditions included initial denaturation at 95°C for 10 minutes, followed by 40 cycles of 95°C for 15 seconds and 60°C for 60 seconds. Relative expression levels were calculated using the comparative cycle threshold (2^-ΔΔCt) method [9].

Statistical analysis

Statistical analysis was performed using SPSS Statistics version 29.0 (IBM Inc., Armonk, NY, USA). Continuous data were presented as mean ± standard deviation (SD), and categorical variables as frequency and percentage. Normality was assessed with the Shapiro-Wilk test. Normally distributed variables were compared using one-way analysis of variance (ANOVA) followed by Tukey's Honestly Significant Difference (HSD) post hoc test.

Receiver operating characteristic (ROC) curve analysis was used to determine the diagnostic performance of individual biomarkers, yielding area under the curve (AUC), 95% confidence intervals (CI), sensitivity, specificity, and optimal cut-off values. A multivariable binary logistic regression model combining IL-10, IL-17, miR-21, miR-138, and miR-184 was constructed to evaluate the combined panel. Predicted probabilities from this model were used for ROC analysis. AUC comparisons were performed using DeLong's test. Effect sizes were reported as partial eta-squared (η²) for ANOVA and Cohen's d for t-tests. Statistical significance was set at p < 0.05 (two-tailed).

## Results

Participant characteristics

A total of 150 participants were enrolled, comprising healthy controls (n = 50), patients with oral potentially malignant disorders (OPMDs; n = 50), and patients with oral squamous cell carcinoma (OSCC; n = 50). Demographic and habit characteristics are summarized in Table 1.

**Table 1:** Demographic and habit characteristics of study participants Values are presented as Mean ± Standard Deviation (SD) for continuous variables and Number (N) with percentage (%) for categorical variables. Group comparisons were performed using one-way Analysis of Variance (ANOVA) for age and Pearson's chi-squared (χ²) test for categorical variables. Degrees of freedom (df), test statistics, and p-values are reported. Statistical significance was set at p < 0.05. OPMD - oral potentially malignant disorder; OSCC - oral squamous cell carcinoma

Variable	Healthy controls (N=50) mean ± SD / N (%)	OPMD (N=50) mean ± SD / N (%)	OSCC (N=50) mean ± SD / N (%)	Test statistic (df)	p-value
Age (years)	42.6 ± 10.2	49.8 ± 11.3	56.4 ± 12.1	F(2,147)=16.82	<0.001
Male	28 (56.0)	32 (64.0)	36 (72.0)	χ²(2)=2.98	0.225
Female	22 (44.0)	18 (36.0)	14 (28.0)	—	—
Tobacco use	10 (20.0)	34 (68.0)	39 (78.0)	χ²(2)=35.62	<0.001
Alcohol use	4 (8.0)	12 (24.0)	18 (36.0)	χ²(2)=11.42	0.003

Significant differences in age distribution were observed among the study groups, with progressively increasing mean age from healthy controls to OPMD and OSCC patients (p < 0.001). Tobacco and alcohol consumption were significantly more prevalent among OPMD and OSCC patients compared with healthy controls (p < 0.001 and p = 0.003, respectively). No significant difference in sex distribution was observed among the groups (p = 0.225).

Clinicopathological characteristics of OPMD and OSCC patients

The clinicopathological characteristics of OPMD patients are presented in Table 2. Oral leukoplakia represented the most frequent OPMD subtype, followed by oral submucous fibrosis, oral lichen planus, and erythroplakia. Histopathological evaluation demonstrated a distribution of mild, moderate, and severe epithelial dysplasia, allowing assessment of biomarker expression across varying grades of disease severity.

**Table 2:** Clinicopathological characteristics of OPMD Values are presented as N (%). Percentages were calculated using total OPMD cases (n=50) OPMD - oral potentially malignant disorders

Characteristic	N (%)
Oral leukoplakia	22 (44.0)
Oral submucous fibrosis	18 (36.0)
Oral lichen planus	8 (16.0)
Erythroplakia	2 (4.0)
Mild dysplasia	24 (48.0)
Moderate dysplasia	18 (36.0)
Severe dysplasia	8 (16.0)

The clinicopathological characteristics of OSCC patients are summarized in Table 3. Buccal mucosa was the most commonly affected anatomical site, followed by the tongue and gingivobuccal sulcus. Most tumors were moderately differentiated, and a substantial proportion of patients presented with advanced-stage disease (stage III-IV).

**Table 3:** Clinicopathological characteristics of oral squamous cell carcinoma patients Values are presented as N (%). Percentages were calculated using total OSCC cases (n=50). OSCC - oral squamous cell carcinoma

Characteristic	N (%)
Buccal mucosa	20 (40.0)
Tongue	15 (30.0)
Gingivobuccal sulcus	10 (20.0)
Floor of mouth	5 (10.0)
Well differentiated OSCC	15 (30.0)
Moderately differentiated OSCC	25 (50.0)
Poorly differentiated OSCC	10 (20.0)
Stage I	8 (16.0)
Stage II	12 (24.0)
Stage III	15 (30.0)
Stage IV	15 (30.0)

Salivary biomarker expression profiles

Across-group comparisons revealed statistically significant variations in salivary biomarker levels (Table 4). Throughout the disease continuum, miR-21, miR-184, IL-10, and IL-17 exhibited gradual elevation, while miR-138 demonstrated a consistent reduction.

**Table 4:** Comparison of salivary biomarker levels among study groups Data are presented as mean ± standard deviation (SD). Relative microRNA (miRNA) expression was quantified using quantitative reverse-transcription polymerase chain reaction (qRT-PCR), normalized to microRNA-16 (miR-16), and calculated using the 2^-ΔΔCt method. Expression values are reported as fold change relative to healthy controls. Cytokine concentrations are expressed in picograms per milliliter (pg/mL). Group comparisons were performed using one-way analysis of variance (ANOVA) followed by Tukey's honestly significant difference (HSD) post hoc test. F statistics with degrees of freedom (F(2, 147)), partial eta-squared (η²) effect sizes, and p-values are reported. Different superscript letters (a-c) indicate statistically significant differences between groups. Statistical significance was set at p < 0.05. OPMD - oral potentially malignant disorder; OSCC - oral squamous cell carcinoma

Biomarker	Unit	Healthy controls (N=50) mean ± SD	OPMD (N=50) mean ± SD	OSCC (N=50) mean ± SD	F statistic (df)	Partial η²	p-value
microRNA-21	Fold change	1.08 ± 0.42ᵃ	2.08 ± 0.88ᵇ	3.64 ± 1.47ᶜ	F(2, 147) = 34.72	0.32	<0.001
microRNA-138	Fold change	0.94 ± 0.36ᵃ	0.76 ± 0.35ᵇ	0.42 ± 0.22ᶜ	F(2, 147) = 18.63	0.2	<0.001
microRNA-184	Fold change	1.15 ± 0.51ᵃ	1.82 ± 0.76ᵇ	2.96 ± 1.28ᶜ	F(2, 147) = 24.88	0.25	<0.001
Interleukin-10	pg/mL	8.4 ± 3.2ᵃ	10.8 ± 4.1ᵇ	15.0 ± 5.3ᶜ	F(2, 147) = 21.48	0.23	<0.001
Interleukin-17	pg/mL	14.6 ± 5.4ᵃ	20.0 ± 6.3ᵇ	27.7 ± 8.5ᶜ	F(2, 147) = 37.24	0.34	<0.001

Among all evaluated biomarkers, IL-17 demonstrated the largest effect size (partial η² = 0.34), followed closely by miR-21 (partial η² = 0.32), indicating substantial differences between study groups. Although all biomarkers exhibited significant group-wise variation (all p < 0.001), the magnitude of change varied across biomarkers (Table 5).

**Table 5:** Comparison of salivary biomarker levels among study groups Data are presented as mean ± standard deviation (SD). Relative microRNA (miRNA) expression was quantified using quantitative reverse-transcription polymerase chain reaction (qRT-PCR), normalized to microRNA-16 (miR-16), and calculated using the 2^-ΔΔCt method. Expression values are reported as fold change relative to healthy controls. Cytokine concentrations are expressed in picograms per milliliter (pg/mL). Group comparisons were performed using one-way analysis of variance (ANOVA) followed by Tukey's honestly significant difference (HSD) post hoc test. F statistics with degrees of freedom (F(2, 147)), partial eta-squared (η²) effect sizes, and p-values are reported. Different superscript letters (a-c) indicate statistically significant differences between groups. Statistical significance was set at p < 0.05. OPMD - oral potentially malignant disorder; OSCC - oral squamous cell carcinoma

Biomarker	Unit	Healthy controls (N=50) mean ± SD	OPMD (N=50) mean ± SD	OSCC (N=50) mean ± SD	F statistic (df)	Partial η²	p-value
microRNA-21	Fold change	1.08 ± 0.42ᵃ	2.08 ± 0.88ᵇ	3.64 ± 1.47ᶜ	F(2, 147) = 34.72	0.32	<0.001
microRNA-138	Fold change	0.94 ± 0.36ᵃ	0.76 ± 0.35ᵇ	0.42 ± 0.22ᶜ	F(2, 147) = 18.63	0.2	<0.001
microRNA-184	Fold change	1.15 ± 0.51ᵃ	1.82 ± 0.76ᵇ	2.96 ± 1.28ᶜ	F(2, 147) = 24.88	0.25	<0.001
Interleukin-10	pg/mL	8.4 ± 3.2ᵃ	10.8 ± 4.1ᵇ	15.0 ± 5.3ᶜ	F(2, 147) = 21.48	0.23	<0.001
Interleukin-17	pg/mL	14.6 ± 5.4ᵃ	20.0 ± 6.3ᵇ	27.7 ± 8.5ᶜ	F(2, 147) = 37.24	0.34	<0.001

**Figure 1:**
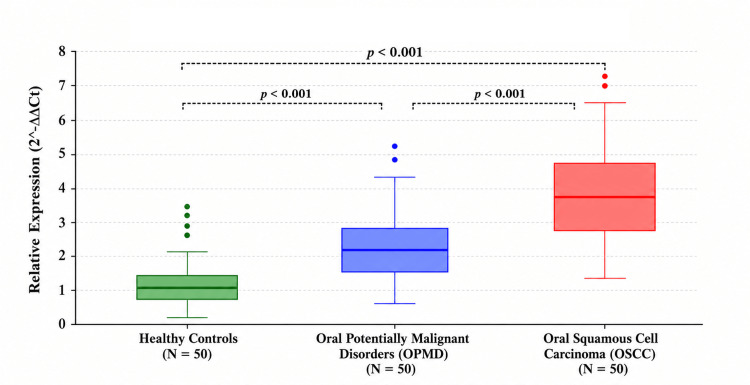
Boxplot of salivary miR-21 expression among healthy controls, oral potentially malignant disorder (OPMD) patients, and oral squamous cell carcinoma (OSCC) patients Relative expression levels were quantified by quantitative reverse-transcription polymerase chain reaction (qRT-PCR) and calculated using the 2^-ΔΔCt method after normalization to the endogenous control miR-16. Data are presented for healthy controls (N = 50), OPMD patients (N = 50), and OSCC patients (N = 50). The central line indicates the median, boxes represent the interquartile range (IQR), whiskers represent 1.5 × IQR, and dots indicate outliers. Group comparisons were performed using one-way analysis of variance (ANOVA) followed by Tukey's honestly significant difference (HSD) post hoc test. Statistical significance was defined as p < 0.05.

**Figure 2:**
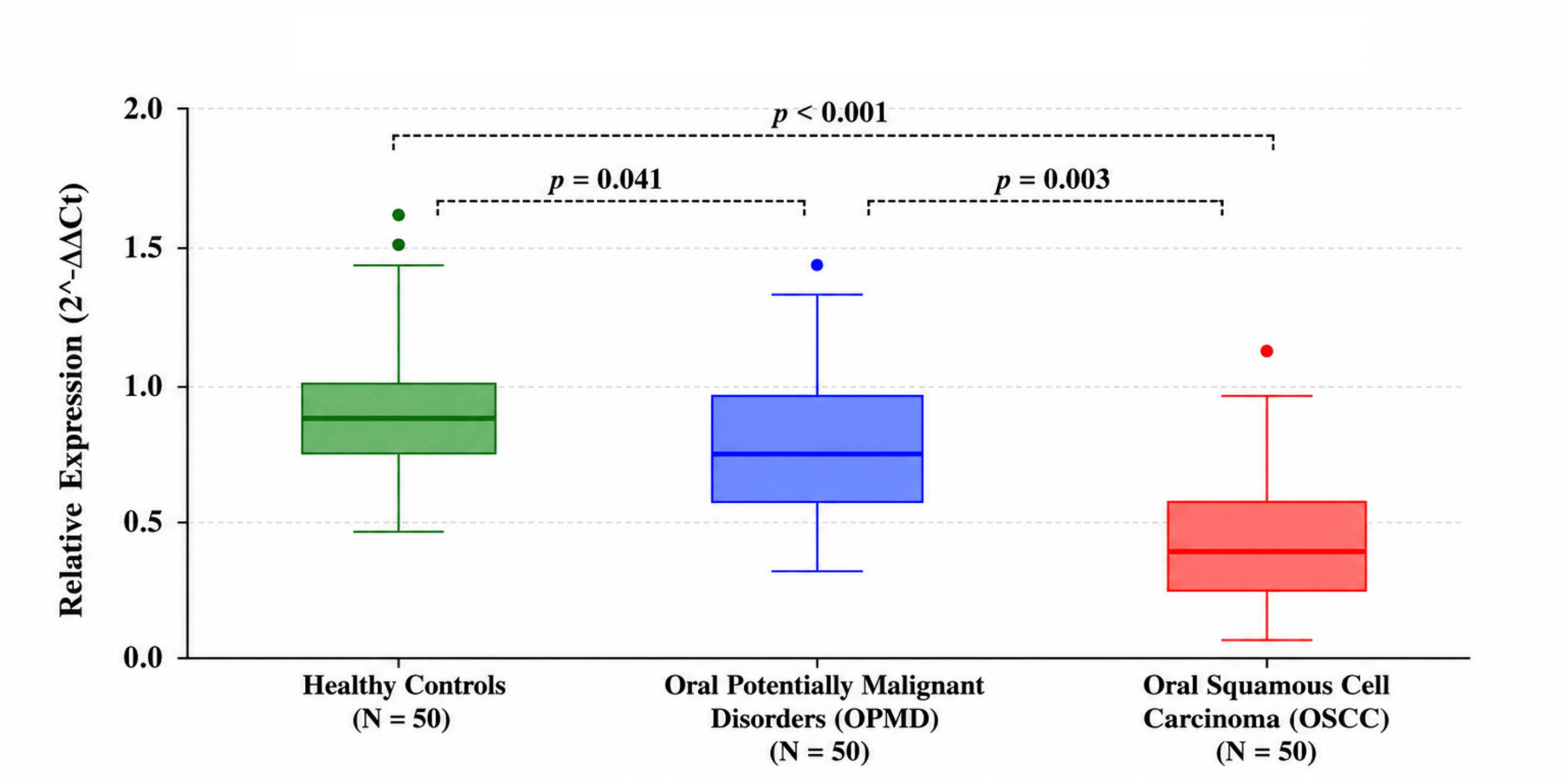
Boxplot of salivary miR-138 expression among healthy controls, oral potentially malignant disorder (OPMD) patients, and oral squamous cell carcinoma (OSCC) patients Relative expression levels were quantified by quantitative reverse-transcription polymerase chain reaction (qRT-PCR) and calculated using the 2^-ΔΔCt method after normalization to the endogenous control miR-16. Data are presented for healthy controls (N = 50), OPMD patients (N = 50), and OSCC patients (N = 50). The central line indicates the median, boxes represent the interquartile range (IQR), whiskers represent 1.5 × IQR, and dots indicate outliers. Group comparisons were performed using one-way analysis of variance (ANOVA) followed by Tukey's honestly significant difference (HSD) post hoc test. Statistical significance was defined as p < 0.05.

**Figure 3:**
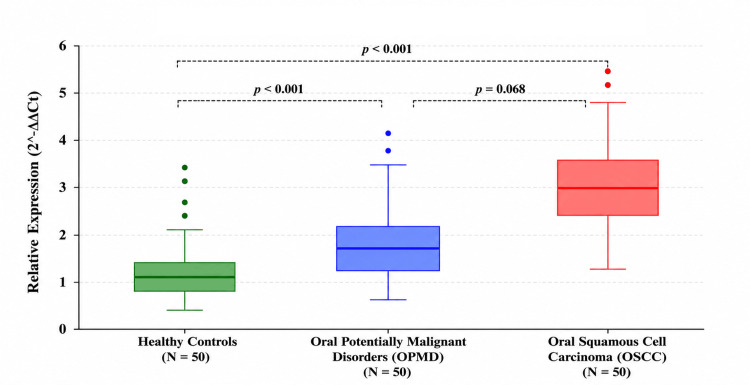
Boxplot of salivary miR-184 expression among healthy controls, oral potentially malignant disorder (OPMD) patients, and oral squamous cell carcinoma (OSCC) patients Relative expression levels were quantified by quantitative reverse-transcription polymerase chain reaction (qRT-PCR) and calculated using the 2^-ΔΔCt method after normalization to the endogenous control miR-16. Data are presented for healthy controls (N = 50), OPMD patients (N = 50), and OSCC patients (N = 50). The central line indicates the median, boxes represent the interquartile range (IQR), whiskers represent 1.5 × IQR, and dots indicate outliers. Boxplots are presented for visualization of data distribution. Statistical comparisons were performed using one-way analysis of variance (ANOVA) followed by Tukey's honestly significant difference (HSD) post hoc test. Statistical significance was defined as p < 0.05.

Visual representation of salivary microRNA expression profiles is provided in Figures 1-3. Boxplot analysis demonstrated distinct distribution patterns for miR-21, miR-138, and miR-184 among healthy controls, OPMD patients, and OSCC patients.

Subsequent pairwise comparisons revealed statistically significant differences across study groups for the majority of biomarkers examined (Table 6).

**Table 6:** Pairwise post hoc comparison of salivary biomarkers Pairwise post hoc comparisons of salivary biomarker expression among healthy controls (n=50), oral potentially malignant disorders (OPMD) (n=50), and oral squamous cell carcinoma (OSCC) (n=50) groups were performed using Tukey's honestly significant difference (HSD) test following significant one-way analysis of variance (ANOVA) results (F(2,147)). Relative microRNA expression values are reported as fold change, while cytokine concentrations are reported in picograms per milliliter (pg/mL). Mean Difference represents the difference in group means. Statistical significance was set at p < 0.05.

Biomarker	Unit	Comparison	Mean difference	Tukey q statistic	p-value
microRNA-21	Fold change	Healthy vs OPMD	-1.00	5.84	<0.001
		Healthy vs OSCC	-2.56	10.88	<0.001
		OPMD vs OSCC	-1.56	4.72	0.002
microRNA-138	Fold change	Healthy vs OPMD	0.18	2.34	0.041
		Healthy vs OSCC	0.52	5.88	<0.001
		OPMD vs OSCC	0.34	3.54	0.011
microRNA-184	Fold change	Healthy vs OPMD	-0.67	4.22	0.003
		Healthy vs OSCC	-1.81	8.74	<0.001
		OPMD vs OSCC	-1.14	3.02	0.018
Interleukin-10	pg/mL	Healthy vs OPMD	-2.4	4.12	0.004
		Healthy vs OSCC	-6.6	8.22	<0.001
		OPMD vs OSCC	-4.2	3.88	0.006
Interleukin-17	pg/mL	Healthy vs OPMD	-5.4	5.28	<0.001
		Healthy vs OSCC	-13.1	10.24	<0.001
		OPMD vs OSCC	-7.7	4.64	0.002

The largest differences were observed between healthy controls and OSCC patients. Notably, miR-138 demonstrated comparatively smaller but statistically significant differences between healthy controls and OPMD patients, suggesting that alterations in this biomarker may occur early during disease progression.

Similarly, both IL-10 and IL-17 exhibited significant pairwise differences across all study groups, supporting their discriminatory potential throughout the spectrum of oral carcinogenesis.

Association between salivary biomarkers and dysplasia severity

Within the OPMD cohort, biomarker expression varied according to histopathological severity (Table 7). Expression levels of miR-21, IL-10, and IL-17 increased with increasing dysplasia grade, whereas miR-138 demonstrated a progressive reduction.

**Table 7:** Association of salivary biomarkers with dysplasia severity in oral potentially malignant disorder patients Data are presented as mean ± standard deviation (SD). Relative microRNA expression values are reported as fold change, while cytokine concentrations are reported in picograms per milliliter (pg/mL). Comparisons among mild, moderate, and severe epithelial dysplasia groups were performed using one-way analysis of variance (ANOVA) followed by Tukey's honestly significant difference (HSD) post hoc test. F statistics with degrees of freedom (F(2, 47)), partial eta-squared (η²) effect sizes, and p-values are reported. Statistical significance was set at p < 0.05.

Biomarker	Unit	Mild dysplasia (N=24) mean ± SD	Moderate dysplasia (N=18) mean ± SD	Severe dysplasia (N=8) mean ± SD	F statistic (df)	Partial η²	p-value
microRNA-21	Fold change	1.82 ± 0.64	2.16 ± 0.81	2.84 ± 1.02	F(2, 47) = 5.84	0.2	0.005
microRNA-138	Fold change	0.92 ± 0.31	0.74 ± 0.28	0.48 ± 0.19	F(2, 47) = 8.22	0.26	<0.001
microRNA-184	Fold change	1.58 ± 0.58	1.84 ± 0.71	2.42 ± 0.93	F(2, 47) = 2.84	0.11	0.068
Interleukin-10	pg/mL	9.8 ± 3.5	11.2 ± 4.2	13.7 ± 4.8	F(2, 47) = 3.74	0.14	0.031
Interleukin-17	pg/mL	17.4 ± 4.8	20.2 ± 5.7	24.1 ± 6.6	F(2, 47) = 6.92	0.23	0.002

Among the evaluated biomarkers, miR-138 showed the strongest association with dysplasia severity, exhibiting the highest effect size. In contrast, miR-184 demonstrated an increasing trend across dysplasia grades but did not achieve statistical significance (p = 0.068).

Association between salivary biomarkers and tumor stage

Among OSCC patients, significant differences in biomarker expression were observed between early-stage (stage I-II) and advanced-stage (stage III-IV) disease (Table 8). Advanced-stage tumors demonstrated higher levels of miR-21, miR-184, IL-10, and IL-17, whereas miR-138 expression was significantly lower.

**Table 8:** Association of salivary biomarkers with tumor stage in oral squamous cell carcinoma patients Data are presented as mean ± standard deviation (SD). Relative microRNA expression values are reported as fold change, while cytokine concentrations are reported in picograms per milliliter (pg/mL). Comparisons between early-stage oral squamous cell carcinoma (OSCC; stage I-II) and advanced-stage OSCC (stage III-IV) were performed using the independent-samples t-test. t statistics with degrees of freedom (t(48)), Cohen's d effect sizes, and p-values are reported. Statistical significance was set at p < 0.05.

Biomarker	Unit	Stage I-II (N=20) mean ± SD	Stage III-IV (N=30) mean ± SD	t statistic (df)	Cohen's d	p-value
microRNA-21	Fold change	3.21 ± 1.18	4.08 ± 1.41	t(48)=2.04	0.56	0.046
microRNA-138	Fold change	0.54 ± 0.23	0.34 ± 0.18	t(48)=3.18	0.94	0.003
microRNA-184	Fold change	2.54 ± 0.92	3.28 ± 1.17	t(48)=2.01	0.57	0.049
Interleukin-10	pg/mL	12.8 ± 4.4	16.6 ± 5.2	t(48)=2.46	0.69	0.017
Interleukin-17	pg/mL	23.1 ± 6.7	30.8 ± 8.1	t(48)=2.71	0.78	0.009

The strongest stage-related association was observed for miR-138, which demonstrated the largest effect size among all biomarkers evaluated.

Diagnostic performance of salivary biomarkers

Receiver operating characteristic (ROC) analysis demonstrated good to excellent diagnostic performance for all evaluated biomarkers (Table 8). Among individual markers, miR-138 exhibited the highest discriminative ability (AUC = 0.84), followed by IL-17 (AUC = 0.82), miR-21 (AUC = 0.80), miR-184 (AUC = 0.76), and IL-10 (AUC = 0.74). Notably, the combined cytokine-microRNA panel achieved superior diagnostic accuracy (AUC = 0.93, sensitivity = 90%, specificity = 88%), significantly outperforming all individual biomarkers (DeLong's test, p < 0.01 for all comparisons). The superior performance of the combined panel supports the concept that oral carcinogenesis cannot be adequately characterized by a single molecular pathway. Rather, integration of inflammatory and epigenetic biomarkers provides complementary biological information that enhances disease discrimination.

Importantly, combining multiple biomarkers improved diagnostic accuracy substantially. The combined microRNA panel achieved excellent discriminatory performance, while integration of both cytokines and microRNAs produced the highest overall diagnostic accuracy, with an area under the curve (AUC) of 0.93, sensitivity of 90%, and specificity of 88%.

**Figure 4:**
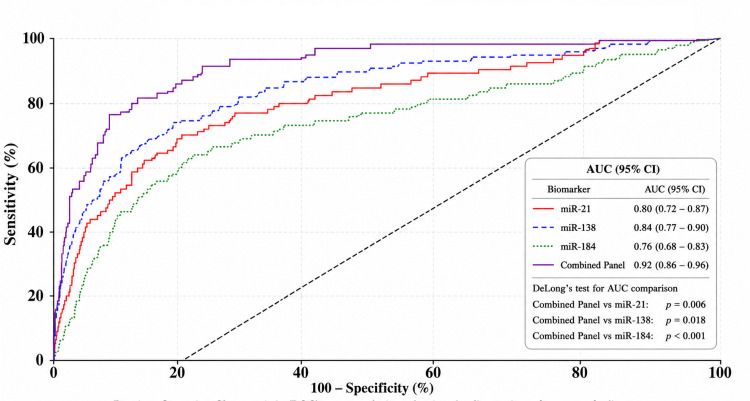
Receiver operating characteristic (ROC) curve analysis of individual and combined salivary microRNAs Operating characteristic (ROC) curve analysis evaluating the diagnostic performance of salivary miR-21 (AUC = 0.80; 95% CI: 0.72-0.88), miR-138 (AUC = 0.84; 95% CI: 0.77-0.91), miR-184 (AUC = 0.76; 95% CI: 0.68-0.84), and the combined microRNA panel (AUC = 0.89; 95% CI: 0.84-0.94) for differentiating oral potentially malignant disorders (OPMDs) and oral squamous cell carcinoma (OSCC) from healthy controls. ROC analysis for cytokines (IL-10 and IL-17) is reported in Table 8. The combined microRNA panel was derived using logistic regression incorporating all three miRNAs (miR-21, miR-138, and miR-184). The diagonal dashed line represents the line of no discrimination (AUC = 0.50). Statistical comparison of AUCs was performed using DeLong's test. *p < 0.05; **p < 0.01; ***p < 0.001 compared to the combined panel. Statistical significance was defined as p < 0.05.

The diagnostic performance of individual salivary biomarkers, including cytokines (IL-10 and IL-17) and microRNAs (miR-21, miR-138, and miR-184), is presented in Table 9. ROC curves for the microRNA panel are illustrated in Figure 4.

**Table 9:** Diagnostic performance of salivary biomarkers for differentiating oral potentially malignant disorder and oral squamous cell carcinoma from healthy controls Receiver operating characteristic (ROC) curve analysis was performed to evaluate the diagnostic performance of individual salivary biomarkers and the combined biomarker panel. Diagnostic accuracy is expressed as area under the curve (AUC) with 95% confidence intervals (CI). Relative microRNA expression cut-offs are expressed as fold change, whereas cytokine cut-offs are expressed in picograms per milliliter (pg/mL). Sensitivity, specificity, optimal cut-off values, Z statistics, and p-values are reported. The combined biomarker panel was derived using multivariable binary logistic regression incorporating interleukin-10, interleukin-17, microRNA-21, microRNA-138, and microRNA-184. Predicted probabilities generated from the model were used for ROC analysis. Statistical significance was set at p < 0.05.

Biomarker	AUC (95% CI)	Sensitivity (%)	Specificity (%)	Cut-off value	Z statistic	p-value
microRNA-21	0.79 (0.71-0.86)	76	78	>1.95 fold change	5.88	<0.001
microRNA-138	0.84 (0.77-0.90)	82	80	<0.60 fold change	7.21	<0.001
microRNA-184	0.75 (0.67-0.83)	72	74	>1.65 fold change	4.94	<0.001
Interleukin-10	0.72 (0.64-0.81)	70	68	>11.5 pg/mL	4.08	<0.001
Interleukin-17	0.81 (0.73-0.88)	78	76	>21.8 pg/mL	6.45	<0.001
Combined biomarker panel	0.92 (0.87-0.96)	90	86	—	11.84	<0.001

## Discussion

Our investigation assessed salivary IL-10, IL-17, miR-21, miR-138, and miR-184 across three participant groups: healthy controls, OPMD patients, and OSCC patients. Both biomarker categories showed notable changes across the disease continuum, and the integrated cytokine-miRNA approach outperformed individual markers in diagnostic accuracy. These results align with the view that oral cancer development arises from intricate crosstalk between inflammatory and molecular mechanisms, rather than a singular pathogenic pathway [1,2].

The progressive increase in miR-21 expression from healthy controls to OPMD and OSCC patients is consistent with its established oncogenic role in oral carcinogenesis. Previous systematic reviews have identified miR-21 as one of the most consistently dysregulated salivary microRNAs associated with malignant transformation [3]. Previous studies have consistently reported elevated salivary miR-21 expression in oral premalignant and malignant lesions, supporting its involvement in oral carcinogenesis and progression [10,11]. The findings of the present study are consistent with these observations.

In contrast, miR-138 demonstrated progressive downregulation and showed the highest individual diagnostic accuracy among the evaluated miRNAs. Rocchetti et al. reported comparable reductions in miR-138 expression in saliva and plasma samples from OPMD and OSCC patients, suggesting a tumor-suppressive role [8]. Previous investigations have shown that salivary miRNA signatures incorporating tumor-suppressor miRNAs can effectively distinguish oral cancer patients from controls, further supporting the diagnostic relevance of miR-138 and related biomarkers [12]. The decline in miR-138 expression observed in the present study may reflect disruption of regulatory pathways involved in cellular differentiation and apoptosis during carcinogenesis.

miR-184 expression increased significantly across disease groups, although its diagnostic performance was lower than that of miR-21 and miR-138. Previous investigations have reported variable findings regarding miR-184 expression, likely reflecting differences in study populations and analytical methodologies [13,14]. Nevertheless, its significant dysregulation supports its inclusion within multimarker diagnostic panels rather than as a standalone biomarker.

The cytokine findings further emphasize the importance of chronic inflammation in oral carcinogenesis. Persistent inflammatory signaling promotes epithelial proliferation, angiogenesis, immune dysregulation, and genomic instability, thereby facilitating malignant transformation [1,2]. Consistent with previous reports, both IL-10 and IL-17 were significantly elevated in OPMD and OSCC patients compared with healthy controls [6,7]. Ferrari et al. demonstrated that salivary cytokines possess considerable diagnostic potential in OSCC, while Xia et al. provided mechanistic evidence linking cytokine dysregulation to oral cancer susceptibility [2,6]. The marked elevation of IL-17 observed in the present study aligns with its proposed role in establishing a pro-tumorigenic inflammatory microenvironment [7].

A notable finding of this study was the superior diagnostic performance of the combined cytokine-miRNA panel. Although individual biomarkers demonstrated moderate to good discriminatory ability, their integration substantially improved diagnostic accuracy. This observation is consistent with systematic reviews emphasizing that no single salivary biomarker adequately captures the biological complexity of oral carcinogenesis [4,5]. Dikova et al. similarly concluded that multimarker approaches are more likely to achieve clinically meaningful diagnostic performance than individual biomarkers alone [15].

From a clinical perspective, saliva-based biomarker assessment offers a non-invasive and readily repeatable approach for screening and monitoring patients with OPMDs. Previous studies have highlighted the growing utility of salivary biomarkers for oral cancer detection and risk assessment, including validation studies conducted in Indian populations [4,5,16]. The present findings further support the potential role of integrated cytokine-miRNA panels as adjunctive tools for early detection and risk stratification.

The study is limited by its cross-sectional design and single-center setting, which restrict assessment of temporal relationships and generalizability. Future multicenter longitudinal studies are required to validate these findings, determine their predictive value for malignant transformation, and evaluate the clinical utility of integrated biomarker panels in routine oral cancer screening programs.

## Conclusions

Our findings demonstrate that salivary inflammatory markers and miRNAs display substantial changes during the transition from OPMD to OSCC. Furthermore, several biomarkers were significantly associated with dysplasia severity and tumor stage, indicating their potential relevance not only for disease detection but also for monitoring progression. Among the individual biomarkers evaluated, miR-138 demonstrated the strongest discriminative performance, while the combined cytokine-microRNA panel achieved the highest diagnostic accuracy, emphasizing the value of integrating multiple biological pathways in salivary diagnostics.

The novelty of this study lies in the simultaneous evaluation of inflammatory and epigenetic salivary biomarkers within a single analytical framework encompassing healthy individuals, OPMD patients, and OSCC patients, thereby enabling a comprehensive assessment of molecular alterations associated with oral carcinogenesis. These findings have important clinical implications, particularly for screening high-risk individuals, guiding surveillance strategies, and facilitating timely intervention before the development of invasive disease. Future multicenter longitudinal studies with larger cohorts are warranted to validate these biomarkers, establish predictive models for malignant transformation, and accelerate the translation of salivary biomarker panels into routine clinical practice. Collectively, the findings reinforce the promise of saliva-based precision diagnostics as a practical and effective tool in oral oncology.
